# A Short and Engaging Adaptive Working-Memory Intervention for Children with Developmental Language Disorder: Effects on Language and Working Memory

**DOI:** 10.3390/brainsci12050642

**Published:** 2022-05-13

**Authors:** Lucy A. Henry, Emma Christopher, Shula Chiat, David J. Messer

**Affiliations:** 1 Division of Language and Communication Science, City, University of London, London EC1V 0HB, UK; emma.christopher@city.ac.uk (E.C.); shula.chiat.1@city.ac.uk (S.C.); 2 Faculty of Wellbeing, Education & Language Studies, Open University, Milton Keynes MK7 6AA, UK; david.messer@open.ac.uk

**Keywords:** working-memory training, intervention, developmental language disorder, children

## Abstract

Recent research has suggested that working-memory training interventions may benefit children with developmental language disorder (DLD). The current study investigated a short and engaging adaptive working-memory intervention that targeted executive skills and aimed to improve both language comprehension and working-memory abilities in children with DLD. Forty-seven 6- to 10-year-old children with DLD were randomly allocated to an executive working-memory training intervention (*n* = 24) or an active control group (*n* = 23). A pre-test/intervention/post-test/9-month-follow-up design was used. Outcome measures included assessments of language (to evaluate far transfer of the training) and working memory (to evaluate near transfer of the training). Hierarchical multiple regression analyses controlling for pre-intervention performance and age found the group to be a significant predictor of sentence comprehension and of performance on six untrained working-memory measures at post-intervention and 9-month follow-up. Children in the intervention group showed significantly higher language comprehension and working-memory scores at both time points than children in the active control group. The intervention programme showed the potential to improve working memory and language comprehension in children with DLD and demonstrated several advantages: it involved short sessions over a short period, caused little disruption in the school day, and was enjoyed by children.

## 1. Introduction

The current investigation concerns a short and engaging adaptive working-memory training intervention that aimed to improve both language comprehension and working memory in children with developmental language disorder. As well as looking at ‘far transfer’ to two measures of language comprehension (sentence comprehension and receptive grammar), the study included six measures of ‘near transfer’ to assess whether the intervention led to gains in working-memory tasks that were not directly trained.

The working-memory system is widely accepted as describing the way information is processed and retained [1] and can be seen as a mental workspace which encompasses several skills used for ‘online’ activities during daily life [1,2]. There are several approaches to understanding working memory [3,4,5,6]; one of the most influential is Baddeley and Hitch’s [7] ‘Multi Component Model of Working Memory’, and its subsequent revision by Baddeley [8]. This model consists of several interacting components, including a limited attentional capacity control system, termed the central executive, which is assisted by two further ‘passive’ components, the phonological loop, for holding speech-based information, and the visuospatial sketchpad, for holding visual and spatial information [9,10]. The central executive relies heavily, but not exclusively, on the frontal lobes [11], and is actively responsible for attentional control, directing and allocating resources in activities when there is a need for the retention of information together with the processing of other information [9,10]. We use the term ‘executive working memory’ (EWM) to refer to working-memory assessments that involve input from the central executive (e.g., concurrent storage and processing) and to interventions that target this component. There also is a fourth component, the episodic buffer, that provides links to long-term memory and some additional multi-modal storage, but this component will not be considered further.

Researchers have emphasised that understanding working-memory development is crucial for helping children maximize their intellectual progress [12]. There are significant associations between the components of the working-memory system and other cognitive processes, especially those related to language [13], literacy [14] and intelligence [15,16] (but see [17]). It is notable that many groups of children with developmental conditions have significantly lower abilities when assessed on the components of working memory compared to children with typical development (TD) who have a similar chronological or sometimes even mental age [1]. Examples of this are: language disabilities [18], dyslexia [19], intellectual disabilities [20], and autism [21].

These associations between working memory and other cognitive processes, as well as the lower working-memory performance of children with developmental conditions, underpin the interest in using interventions to improve working memory and related neuropsychological abilities. An investigation of a computerised working-memory intervention targeting the central executive found that, following training, performance on components of the working-memory system improved, and importantly, non-verbal intelligence also improved [22]. These effects were reported for children with attention deficit hyperactivity disorder and for a small sample of adults with TD [22]. This initial study was followed by further investigations of this claim, which have been summarised in subsequent systematic reviews and meta-analyses. In general, these reviews have concluded that training can result in near transfer, involving an increase in other working-memory abilities that were not part of the intervention procedure, although the evidence for the persistence of these effects is variable. The reviews have also concluded that interventions generally do not result in far transfer to other cognitive abilities such as those associated with mathematics, intelligence, literacy, or language.

However, systematic reviews to date have usually been concerned with computer-based interventions that attempt to improve at least one component of the working-memory system [23,24,25,26] or non-computerised interventions involving mainly children with typical development [27]. Consequently, although these reviews suggest that working-memory training does not result in far transfer, their focus has not been on non-computerised interventions targeting children with developmental conditions.

It remains important to understand how working-memory interventions might benefit children with developmental conditions. Rowe et al. [27] emphasised the potential importance of exploring working-memory training interventions that target executive skills with children who have language or other disabilities. This is particularly important as a meta-analysis by Peijnenborgh et al. [28] of working-memory interventions with children who have developmental conditions showed that previous research had focussed mainly on children with attention deficit hyperactivity disorder (10/13 studies), or the type of developmental condition was not specified. In addition, several investigations since 2018 challenge the pessimistic conclusions reached in the earlier reviews of this topic. For example, a single case experimental design investigation involving seven children with working-memory difficulties [29] used the Cogmed computer-based intervention [30]. This study found near-transfer effects for all children, and far transfer in language, reading, or mathematics for three children with ‘convincing but modest training effects across multiple measures’ [22] (p. 1).

Several recent investigations of working-memory interventions have concerned children with developmental language disorder (DLD) or a related diagnosis. DLD is a persistent neurodevelopmental condition, involving a primary difficulty with language that is unexplained by any other syndrome or circumstances [31,32]. DLD is estimated to have a prevalence of 7% [33].

Newly emerging evidence suggests a more promising role for working-memory interventions in children with DLD. For example, in a study using a pre-test/intervention/post-test design, Holmes et al. [34] used the Cogmed working-memory intervention with a sample of children who had low language ability. They identified significant training gains for several working-memory components and a far-transfer effect to performance IQ, although this effect was non-significant with Bonferroni corrections. Acosta, Hernandez and Ramirez [35] also used a pre-test/intervention/post-test design, developing a working-memory training which targeted short-term verbal memory and EWM in both verbal and visuospatial domains, delivered using a combination of individual and group sessions. They found significant training gains in all components of the working-memory system for children with language difficulties, as well as far transfer to a lexical–semantic task; they also noted greater gains in their group with language difficulties than in a comparison group with TD. However, neither of these studies included an untreated or active control group, which would provide stronger evidence in favour of the intervention [24].

Acosta-Rodríguez, Ramírez-Santana, and Hernández-Expósito [36] used a comprehensive multi-technique non-computer classroom-based intervention that included executive working-memory training as one element. They included children with DLD and TD, allocating half of the children in each group to receive the intervention and half to receive no treatment. Acosta-Rodríguez and colleagues [36] reported immediate post-intervention improvement on several assessments of language comprehension, verbal EWM and semantic fluency in trained children with DLD compared to the untreated DLD control group. The children with DLD receiving the intervention made greater gains than the equivalent typical group. In a single case experimental design, Maleki Shahmahmood et al. [37] used a mixed non-computer and computer-based intervention with 10 Iranian children who were identified with a primary language impairment. The intervention focused on phonological short-term memory and EWM, as well as suggestions about strategies. Following training over five weeks, increases in working memory and morpho-syntactic abilities were reported. A subsequent five-week language intervention also increased morpho-syntactic abilities, but gains were not detected for working memory, suggesting an effect of working-memory training on language, but not vice versa [37].

In two other investigations, far-transfer effects of working-memory training have been reported on syntax with French children who have DLD [38,39]. Stanford et al. [38] used iPad or computer-based training (Magic Memory) in groups with DLD and typical development (TD). They also included active control groups who followed an alternative general scholastic training without working-memory content, and randomly allocated their participants to each condition. The training process involved practising five verbal working-memory tasks assessing two components of the working-memory system (verbal short-term memory and EWM). These working-memory training tasks were adaptive, such that as the child’s performance improved or declined, the task difficulty was adjusted to match the child’s current performance. Improvements in both aspects of working memory were found immediately post-intervention for DLD and TD groups. Furthermore, for the DLD group there were improvements in syntax involving third-person accusative clitics, which are often seen as a marker for DLD in French-speaking children [38]. Delage et al. [39] used the same Magic Memory intervention, reporting improved working memory in DLD and TD groups compared to active control groups immediately post-intervention, with additional far-transfer gains on sentence repetition and complex syntax in the DLD intervention group. Therefore, the findings reported since 2018 are not only promising support for working-memory interventions for children with DLD, but also suggest that children with DLD may gain more from working-memory interventions compared to individuals with TD [24,34,35,36,38,39,40,41].

The present investigation of the effectiveness of an executive working-memory training intervention for children with DLD builds on these findings. We focussed on children with DLD because of the suggestion that children with developmental conditions may be more likely to benefit from working-memory interventions, as there is more room for improvement. Children with DLD are known to have difficulties with working memory, which encompass both short-term and executive working memory [35,42,43,44,45]. A face-to-face rather than a computer-based intervention was chosen because, when the study was planned, meta-analyses suggested computer-based forms of intervention were not effective [24,26]. The intervention targeted EWM skills, based on previous evidence that this is the common feature of effective working-memory interventions [27], and evaluated far-transfer effects to receptive grammar and language comprehension skills. It was predicted that the intervention would improve receptive grammar abilities, given evidence reported above that working-memory interventions can improve morpho-syntax in children with DLD, possibly by increasing information-processing abilities. It was also predicted that the intervention would improve language comprehension, based on previous findings and work by Diamond [46] and others [47] who emphasise the strong links between language comprehension and EWM, both of which require the concurrent processing and retention of continuous input that unfolds over time. In this context, Melby-Lervåg et al.’s [24] report of a small significant effect of working-memory training on reading comprehension (and arithmetic) is interesting, although when they excluded studies that had a significant decline pre- to post-intervention in relevant control groups, the effect was not regarded as noteworthy.

The current intervention followed a programme used by Henry et al. [48], who reported promising longer-term findings for reading comprehension in children with TD. This programme comprised a relatively short, face-to-face, enjoyable, adaptive (an important feature of effective interventions [49]) intervention targeting EWM skills. The intervention employed both verbal and non-verbal EWM tasks as recommended by Danielsson et al. [50], who only found significant training effects to untrained working-memory measures in samples with a developmental condition (intellectual disabilities) when a mixed working-memory approach was adopted that included both verbal and visuo-spatial components. In the current study, participants were assessed before intervention, and re-assessed immediately post-intervention and nine months later, evaluating maintenance over a longer period than any previous study of children with DLD.

The overall research question was: For children with DLD, are there immediate and longer-term effects of EWM training? We assessed immediate and longer-term effects for directly trained EWM tasks, working-memory tasks that were not trained (near transfer) and language comprehension (far transfer).

## 2. Materials and Method

### 2.1. Participants

Fifty-one participants aged six to ten years with language difficulty as a primary need were recruited from six mainstream primary schools (four with a specialist language provision) across Dorset and Hampshire (UK). Study inclusion criteria were: age 6–11 years, language difficulty identified as a primary need (and two or more language scores at or below one standard deviation of the mean on screening), nonverbal IQ of 70 or above, no formal diagnosis of autism, and English as a first spoken language at home.

The study was conducted according to the guidelines of the Declaration of Helsinki and approved by the relevant university Ethics Committee. Written informed consent was sought and obtained from all parents/guardians of participating children, and verbal assent was obtained from the children themselves. Schools recommended suitable children, and following the consent process, screening tasks were undertaken. These included: the four core subtests from Clinical Evaluation of Language Fundamentals 4 (CELF-4-UK) [51], the Wechsler Abbreviated Scale of Intelligence—Second Edition (WASI-II) [52] Block Design and Matrix Reasoning subtests to obtain a measure of non-verbal IQ (Perceptual Reasoning Index), establishing that the child had no formal diagnosis of autism through discussion with the school (to ensure any language difficulty identified could not be related to the autism diagnosis), and ensuring the child’s first language at home was English (to ensure any language difficulty identified could not be due to the child speaking English as an additional language). Four children were excluded: two children had Perceptual Reasoning Index scores below 70, one was undergoing an autism assessment at the time of the intervention, and one did not speak English as a first language. Forty-seven children (mean age = 8 years 0 months, SD = 15 months; range = 6; 2 to 10:6) fulfilled the inclusion criteria.

### 2.2. Random Allocation of Children to Groups

Children who fulfilled the study inclusion criteria were randomly assigned by an independent colleague to one of two groups, a working-memory training intervention group (*n* = 24) or an active control group (*n* = 23). Excluding the researcher, all other individuals including staff at the schools were blind to this random allocation. The researcher was aware of the participant’s group membership as practical limitations made it impossible to conduct a double-blind experiment. Screening scores for both groups are given in Table 1, together with independent-samples *t*-tests for group differences (effect sizes, Cohen’s *d*, with 95% confidence intervals). No significant group differences on the screening variables were found, although there was a tendency for the active controls to show numerically lower language scores.

### 2.3. Screening Tasks

The CELF-4-UK [51] was administered to determine whether language difficulty was a primary need, as this test is commonly used amongst speech and language professionals. Four language subtests, ‘Concepts and Following Instructions’, ‘Word Structure’, ‘Recalling Sentences’, and ‘Formulated Sentences’, were administered to all participants (test–retest reliabilities for CELF-4-UK subtests range from 0.71 to 0.86).

Concepts and Following Directions assesses the child’s ability to interpret, recall, and execute oral commands. The child listens to a set of instructions and points to the relevant pictures, with options presented visually. For example, a child may be asked to ‘point to a fish next to a house’ or ‘point to all the shoes on the top row’. The commands increase in length and complexity and the task stops after seven consecutive scores of zero. Word Structure assesses the knowledge of grammatical rules using a sentence-completion task. Various grammatical rules are presented (irregular plurals, possessive pronouns, future tenses and irregular past tenses), and the child completes an orally presented sentence related to an illustration, e.g., ‘Here is one book. Here are two _____’. There was no discontinuation point and all children were administered 32 sentences. Recalling Sentences evaluates a child’s ability to recall and produce sentences varying in length and syntactic complexity. Orally presented sentences are repeated back by the child and scored for errors. Full recall with no errors gains a score of three, one error a score of two, two to three errors a score of one and more than four errors a score of zero. After five consecutive scores of zero, the subtest was discontinued. Formulated Sentences required the child to formulate semantically and grammatically correct sentences using a target word to describe a given picture. Depending on the grammatical and semantic correctness of a sentence, a child was given a score between zero and two for each item. If a child obtained five consecutive scores of zero, the subtest was discontinued.

The WASI–II [52] assessed non-verbal IQ using the Block Design and Matrix Reasoning subtests to obtain a Perceptual Reasoning Index (PRI) score. Block Design required a child to analyse and reproduce abstract visual stimuli. There were 13 items, the first four required the design to be reproduced from a picture stimulus and a previously constructed model, which the child observed being made by the researcher, and the remaining items from only a picture stimulus. Depending on the completion time, the child was allocated a score from zero to six, and if two consecutive scores of zero were recorded, the subtest was discontinued. Matrix Reasoning required a child to view an incomplete series of matrices and choose one from five options to complete the set. Children were scored either one point for a correct answer or zero for an incorrect answer and the subtest was discontinued after three consecutive scores of zero. The test–retest reliability for the PRI ranges from 0.86 to 0.87.

### 2.4. Outcome Measures at Pre-Intervention, Post-Intervention and 9-Month Follow-Up

All children were administered outcome assessments before the working-memory intervention commenced, immediately after 18 intervention sessions were completed and at a 9-month follow-up. Outcome measures related to direct training effects (the two trained executive working-memory tasks), far-transfer effects (measures of sentence comprehension and receptive grammar), and near-transfer effects (short-term and executive working-memory tasks that were not trained). Raw scores are reported for all outcome measures as standardised tests were not available for some tasks (Odd One Out, Pattern Span), and age was controlled in all relevant analyses. See Table 2 for mean scores in both groups at all time points.

#### 2.4.1. Direct Effects

Listening recall from the Working-Memory Test Battery (WMTB-C) [53] was administered to assess verbal EWM. The task required the child to listen to an orally presented sentence, say whether the sentence was true or false (e.g., ‘Dogs have tails’), and then recall the final word of the sentence (‘tails’). It began with two practice trials. Following this, a block of six trials with a list length of one (i.e., one sentence) was presented. If the child obtained at least four out of six trials correct, the task increased in difficulty by presenting two sentences (judgements were made for each sentence, in turn, and then the two final words recalled). Two practice items were administered, followed by a block of six trials at list length two. Again, if four trials out of six were correct, list length increased to three items and so on until fewer than four correct items in a block were achieved, at which point the test was discontinued. If the first four trials in any block were correct, credit was given for trials 5 and 6, and the task moved up a level. Total trials correct scores were used as outcome measures, and span scores (highest list length achieved successfully) were used for determining the starting point for the working-memory intervention. Test–retest reliability ranges from 0.38 to 0.84.

The Odd One Out Span task [54] assessed a child’s EWM in the visuospatial domain. This task was slightly adapted to be administered on a tablet using a PowerPoint presentation, and the numbers of trials increased to ensure sensitivity at low performance levels. The researcher read through the instructions with the child, and the task began with two practice trials. Each trial showed three black and white images of hard-to-name, but simple forms, presented in the middle of the screen in a horizontal row. The child was asked to point to the ‘odd one out’ (one item was slightly different to the other two). Following this, the child was shown an empty response grid containing three boxes, horizontally arranged to match the presentation screen, and asked to recall the spatial location of the odd one out by pointing to the correct location on the response grid (left, middle or right). The formal task began with six trials at a list length of one. If the child obtained four or more trials correct, the child was moved up to the next list length of two, i.e., two odd-one-out judgements, in turn, and a response screen with two empty grids, one on top of the other, for recalling the two spatial locations. The task proceeded with longer list lengths, provided four out of six trials were correct. If fewer than four trials out of six were correct, the task was discontinued. Total trials correct were used as the outcome measure, and span scores (highest list length achieved successfully) were used for determining the starting point for the working-memory intervention. The reliability for the original version of this task is 0.80 [54].

#### 2.4.2. Far Transfer

The Sentence Comprehension subtest from the Assessment of Comprehension and Expression (ACE) [55] was administered to evaluate the ability to understand sentences of increasing length and complexity, requiring the child to decode verbal concepts of quantity, space, time, and emotion. The task has a ‘broad floor’ that allows even children with very low language abilities to succeed on some items [55]. For many items, the child was shown four pictures on a page which were all similar, but not identical. Each picture had a slight variation to the theme relating to a sentence, which was read aloud to the child, e.g., ‘The helicopter flew above the clouds’. After hearing the sentence, the child was asked to choose which picture fit the sentence best. For other items, children were presented with one or two sentences supported by a picture, sometimes with a set of printed words around the picture, and asked a question related to these. Responses required either a spoken word or a finger-point to one of the items in the picture or printed words, for example, from a multiple choice of words for feelings ‘sad, hungry, cheerful, sleepy’ that are spoken by the tester and printed on the page. The subtest had 35 items, which were all administered as there was no discontinuation point. Raw scores were used. The test–retest reliability is 0.61 and internal consistency is 0.66.

The Test for Receptive Grammar Version 2 (TROG-2) [56] measures the understanding of grammatical constructs. The test covers 20 different constructs, each represented by a block of four items, totalling 80 items. For each item, the child was shown four pictures on one page. One of these pictures represented a target sentence that was read to the child, whereas the other three pictures acted as foils that depicted a sentence that was altered by a grammatical or lexical element. A discontinuation rule applied if the child answered five consecutive items incorrectly. Raw scores in terms of the number of blocks passed were used. The parallel form reliability for standardised scores on this test is 0.71.

#### 2.4.3. Near Transfer

Four tasks assessed short-term working-memory abilities (two measures of verbal short-term memory and a measure each of spatial and visual short-term memory). For five of the measures (all from the WMTB-C [53]), the tasks proceeded by presenting blocks of six trials, beginning with list lengths of one item. If four trials out of six were correct within a block, the list length increased by one, and so on, until fewer than four correct items in a block were achieved, at which point the test was discontinued. If the first four trials in any block were correct, credit was given for trials 5 and 6, and the task moved up a level. Total trial correct scores were used as outcome measures for all five of the following tasks to ensure comparability with the non-standardised measures (which took the same format).

Digit Recall (WMTB-C) assessed verbal short-term memory and involved the experimenter reading out a list of numbers which the child repeated back in the correct order. The test–retest reliability for Digit Recall ranges from 0.81 to 0.82.

Word List Recall (WMTB-C) assessed verbal short-term memory and involved the experimenter reading out a list of one syllable words which the child repeated back in the correct order. The test–retest reliability for Word List Recall ranges from 0.64 to 0.80.

Block Recall (WMTB-C) assessed spatial short-term memory and involved showing the child a board with nine randomly spread out identical raised cubes (3 cm × 3 cm, coloured grey). The researcher tapped a series of one or more spatial positions on the blocks and asked the child to recall the positions by tapping the same sequence immediately afterwards. The test–retest reliability for Block Recall ranges from 0.43 to 0.63.

Counting Recall (WMTB-C) assessed executive working memory and required the child to count, out loud, the number of dots that were presented to them in a stimulus book. After counting the dots, the researcher turned the page of the stimulus book, and the child was asked to recall the number of dots they previously counted. Trials started with a single page of dots and were given in list lengths of six, as per the other tests. The test–retest reliability for Counting Recall ranges from 0.48 to 0.74.

Backward Digit Recall (WMTB-C) assessed executive working memory and involved the experimenter reading out a list of numbers, which the child had to repeat backwards. The test–retest reliability for Backward Digit Recall ranges from 0.53 to 0.71.

A measure of the Pattern Span was developed, based on that of Della Sala et al. [57], to assess visual short-term memory using a similar procedure to the WMTB-C tasks. Pattern span required the child to look at the position of a coloured-in red square on a 2 × 2 black-and-white grid, and then recall that position on an identical but empty black and white grid. This task had three trials per block, and each block comprised one list length (list length corresponded to the number of red squares) for one specific grid size, with list lengths and grid sizes systematically increasing in difficulty as the task progressed. The first three trials (block 1) required the child to recall one red square on a 2 × 2 grid. The second three trials (block 2) required the recall of two red squares on a 2 × 2 grid. Next, the grid size was increased to 2 × 3, with three trials recalling two red squares (block 3). Next, the number of squares was increased with three further trials recalling three red squares (block 4). The grid size then increased again to 3 × 3, and there were a further three trials recalling three red squares (block 5), and three trials recalling four red squares (block 6). This repeating pattern of grid size increases and list length increments continued in blocks of three trials, until the last set of trials required the child to recall seven red squares on a 4 × 5 grid. The researcher discontinued the task if the child did not score four or more correct in any set of six trials with the same list length (i.e., number of red squares to recall, regardless of grid size). Scores were the total trials correct over all blocks. Internal consistency was calculated by determining separate trial correct scores for all trial 1 s, all trial 2 s, and all trial 3 s within each block (for a similar approach see [5]). Correlations were *r* = 0.71 between trial 1 and trial 2 scores, *r* = 0.79 between trial 1 and trial 3 scores, and *r* = 0.82 between trial 2 and trial 3 scores).

### 2.5. Study Procedure

For the screening phase, the researcher visited the child at school and administered the CELF-4-UK subtests and the WASI-II Perceptual Reasoning Index tasks. Following random allocation to working-memory training and active control groups, children completed assessments in working memory (eight tasks), sentence comprehension and receptive grammar (measures of reading accuracy and comprehension were also administered, but due to floor effects, they are not considered further). Next, children received the working-memory training intervention (see below) and, following the intervention, all outcome measures were administered again. After a delay of around 9 months, outcome measures were administered once more to assess maintenance.

Those assigned into the working-memory training group undertook the full intervention protocol over a six-week period. During the intervention, all children were visited three times a week within their school setting at the same time each week, in a quiet area either in a separate classroom, library area or intervention room. Each child spent around 10 min with the researcher administering 11 trials similar to those in the Listening Recall task [53] and 11 trials similar to those in the Odd One Out Span task [54]. The task was adaptive, reflecting the child’s performance from trial to trial; therefore, the researcher continuously monitored performance, first to determine previous span levels on each task, second to ensure that the task administered first alternated from session to session, and third to record every span performance score within each training session. The researcher always checked that the child was aware of the task instructions before administering the 11 trials of each training task. The same procedure occurred for the active control group; however, the working-memory element was removed from the training tasks. In this way, the inclusion of an active control group should rule out training benefits accruing from global stimulation, individual time with the researcher, or maturation [23,38]. All children in both groups enjoyed the sessions and were happy to attend each time. In the event of school absence, the researcher saw the child on another day such that all children completed all 18 training sessions. Further details about the executive working-memory training tasks are provided below.

#### 2.5.1. Listening Recall Training Task

This training task (adapted from Listening Recall, WMTB-C [53]) focused on the child’s verbal EWM. A different set of listening recall stimuli to the outcome assessment materials was used to eliminate practice effects and overlap.

The listening recall training materials comprised six sets of sentence stimuli for every list length between one and seven, which could be drawn from during the training session to produce trials at the appropriate level. The first training trial began at the child’s span level, established from pre-intervention testing. If the span level was previously zero, the researcher started at span level one. The researcher orally presented the requisite number of sentences depending on the start span level, each was judged as true or false by the child, and the child attempted to recall the final word from each sentence. Two trials at the initial span level were presented. If both trials were answered correctly, the researcher moved the child up one span level, increasing the task difficulty. If both trials were answered incorrectly, the researcher lowered the span level by one (or if already at one continued with this level). If the child answered one trial correctly and one trial incorrectly, the span level remained the same for a further two trials. The training continued in this format until the child answered 11 trials. At the end of the 11 trials, the researcher calculated the most commonly administered span level and recorded this on the record form so that this would be the start level for the next training session. The process was repeated for the next 17 training sessions of the working-memory intervention.

The children assigned to the active control group were also administered 11 trials per session but were not required to recall the final word of the sentence. Thus, the active control group just needed to identify whether the sentence was true or false, removing any working-memory requirement to the task.

#### 2.5.2. Odd One Out Span Training Task

**Figure 1:**
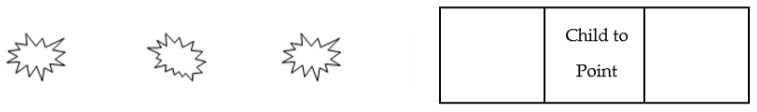
Example of ‘Odd One Out’ Card and Response Card.

This training task (adapted from Odd One Out Span [54]) focused on the child’s visuospatial EWM and was based on the Odd One Out task. A different set of odd-one-out stimuli to the outcome assessment materials was used to eliminate practice effects and overlap. The task was presented with paper-based materials, rather than on a tablet computer, but otherwise had the same format as the Odd One Out task. Odd-one-out stimuli were each presented as black-and-white line drawings on 10 cm × 30 cm white cards. The response card depicted one or more blank horizontal grids, each containing three empty boxes. The child was asked to remember the spatial location of the ‘odd one out’ items by pointing to the correct position/s on the grid (see Figure 1 for examples).

As for the Listening Recall training task, the first training trial began at the child’s span level, established from pre-testing, unless the span level was previously zero, in which case, the researcher started at span level one. Again, the working-memory training was designed to be adaptive, so it increased in difficulty if the child’s memory span performance increased, stayed at the same difficulty if the level was exactly at the child’s level, or decreased in difficulty if span had been exceeded. At the end of the 11 training trials, the most frequently presented list length was carried over to the next session as the ‘starting point’. The process was repeated for the next 17 training sessions of the working-memory intervention.

Children who had been assigned to the active control group were also administered 11 ‘Odd One Out’ trials but made the ‘odd-one-out’ judgment only, i.e., they were not required to recall the spatial locations. For the active control, therefore, the same materials were used, and the same interaction was included with the researcher as for the intervention, removing any working-memory requirement to the task.

## 3. Results

### 3.1. Pre-Test Scores

Mean raw scores for pre-intervention, post-intervention and 9-month follow-up on all outcome measures are given in Table 2. All scores for working-memory tasks represent total trials correct as these scores were comparable across all tasks (standardised and non-standardised), and in some cases, standardised scores were unavailable due to low performance. For Sentence Comprehension, we report raw scores; and for Receptive Grammar, total blocks passed.

There were no significant differences between groups on any pre-test scores (see Table 2 for means and SDs):

- Listening Recall, *t*(45) = −0.23, *p* = 0.819 (*d* = −0.07, 95%CI −0.60, 0.51);
- Odd One Out, *t*(45) = 0.28, *p* = 0.782 (*d* = 0.08, 95%CI −0.49, 0.65);
- Sentence Comprehension, *t*(45) = 1.17, *p* = 0.25 (*d* = 0.34, 95%CI −0.24, 0.91);
- Receptive Grammar, *t*(45) = 1.62, *p* = 0.113 (*d* = 0.47, 95%CI −0.11, 1.05);
- Digit Recall, *t*(45) = 0.82, *p* = 0.415 (*d* = 0.24, 95%CI −0.34, 0.81);
- Word List Recall, *t*(45) = 1.90, *p* = 0.064 (*d* = 0.56, 95%CI −0.03, 1.14);
- Block Recall, *t*(45) = 0.37, *p* = 0.711 (*d* = 0.11, 95%CI −0.46, 0.68);
- Pattern Span, *t*(45) = 0.74, *p* = 0.465 (*d* = 0.22, 95%CI −0.36, 0.79);
- Counting Recall, *t*(45) = 0.80, *p* = 0.427 (*d* = 0.23, 95%CI −0.34, 0.80);
- Backwards Digit Recall, *t*(45) = 0.87, *p* = 0.389 (*d* = 0.25, 95%CI −0.32, 0.83).

Scores for Sentence Comprehension and Receptive Grammar were numerically lower in the active control group, in line with the language screening scores. Therefore, all analyses of outcome measures controlled for pre-test performance so that any differences between groups could be attributed to the intervention and not initial levels of performance [58].

### 3.2. Approach to Analyses

Hierarchical multiple regression was undertaken to examine whether training group (working-memory training vs. active control) was a significant predictor of post-intervention and 9-month follow-up outcome scores. In all regressions, pre-intervention scores for the relevant outcome measures and age in months were controlled at step one. Controlling for pre-intervention outcome scores was a conservative approach as it ensured that any initial variations in performance across participants and groups were taken into account [58]. Age was also controlled as we were using raw scores uncorrected for age. The intervention group, as a dummy variable, was entered at step two of each regression to assess the effects of the intervention.

For all regression analyses, key statistical checks were carried out (Durbin–Watson, tolerance and VIF statistics, Cook’s and Mahalanobis distances, standardised DFBeta, leverage values, plots of standardised residuals, predicted standardised values, standardised residuals and partial plots). In some regression analyses, one child in the working-memory training group improved on an outcome measure more than the model predicted, resulting in a high standardised residual (outlier). When this occurred, regressions were re-run after removing the relevant case, but as there were never any substantive differences in findings, original analyses are reported.

### 3.3. Direct Effects (Listening Recall and Odd One Out)

**Figure 2:**
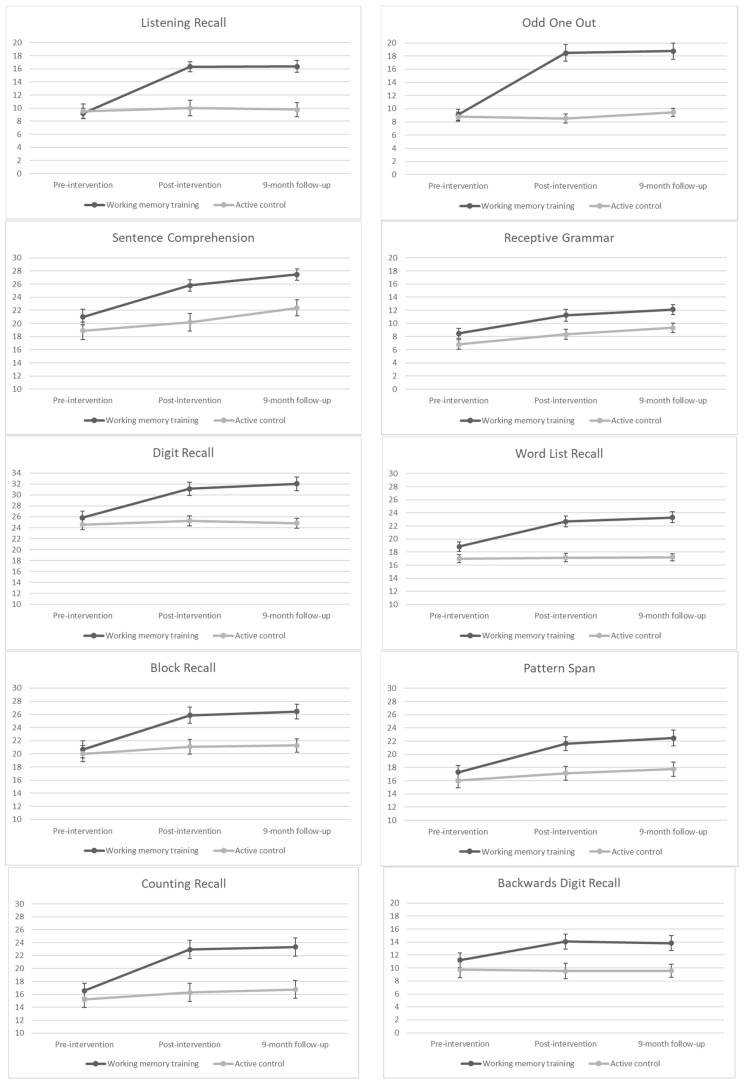
Graphs illustrating raw scores on all outcome measures (with standard error bars) at pre-intervention, post-intervention and 9-month follow-up for both groups (working-memory training and active control).

The first analyses assessed whether training intervention effects were present on the two trained executive working-memory tasks. As four separate regression analyses were conducted (two directly trained executive working-memory tasks at two time points), Bonferroni corrections were made to the significance levels for the overall models (*p* < 0.01). See Table 3 for summary regression information and Figure 2 for graphs illustrating pre-intervention, post-intervention and 9-month follow-up scores for each group.

For post-intervention Listening Recall, at step one of the regression, the pre-intervention scores and age accounted for 44.3% of the variance. Examination of the standardised beta-values indicated that only the pre-intervention score was a significant individual predictor; this was not unexpected as pre-intervention scores are an important predictor for scores after intervention. In step two, adding group produced a significant change in *R*^2^ (∆*R*^2^); here, group accounted for a further 33.8% of the variance. The final model was significant, *F*(3, 43) = 51.06, *p* < 0.001, predicting 78.1% of the total variance (Adj. *R*^2^ 0.766). At 9-month follow-up, pre-intervention scores and age accounted for 44.9% of the variance at step one, with only pre-intervention score as a significant individual predictor. Adding group at step two again produced a significant change in *R*^2^ (∆*R*^2^), with group accounting for a further 35.8% of the variance. The final model was significant, *F*(3, 43) = 59.89, *p* < 0.001, predicting 80.7% of the total variance (Adj. *R*^2^ 0.793).

The training group differences on Listening Recall were large, even after controlling for pre-intervention score and age. Inspection of unstandardised beta values (B) in Table 3 shows that, holding age and pre-intervention score constant, being in the active control group rather than the working-memory training group meant that the Listening Recall trials correct scores were on average 6.61 points lower (effect size *f*^2^ = 0.51) at post-intervention and 6.85 points lower (*f*^2^ = 0.56) at 9-month follow-up. Note: for Cohen’s *f*^2^ [59], the effect sizes are *f*^2^ = 0.02 small; *f*^2^ = 0.15 medium; *f*^2^ = 0.35 large.

For post-intervention Odd One Out, at step one, the pre-intervention scores and age accounted for 30% of the variance, with the pre-intervention score representing a significant individual predictor. Adding group at step two produced a significant change in *R*^2^ (∆*R*^2^), accounting for a further 48.3% of the variance; here, age became a significant predictor alongside pre-intervention score and group. The final model was significant, *F*(3, 43) = 51.58, *p* < 0.001, predicting 78.3% of the total variance (Adj. *R*^2^ 0.767). At 9-month follow-up, pre-intervention scores and age accounted for 31% of the variance, with pre-intervention score a significant individual predictor. Adding group at step two produced a significant change in *R*^2^ (∆*R*^2^), accounting for a further 47.2% of the variance; here, age again became a significant predictor alongside pre-intervention score and group. The final model was significant, *F*(3, 43) = 51.47, *p* < 0.001, predicting 78.2% of the total variance (Adj. *R*^2^ 0.767).

The training group differences for Odd One Out were very large, even after controlling for pre-intervention score and age. Inspection of unstandardised beta values in Table 3 shows that, holding age and pre-intervention score constant, being in the active control group rather than the working-memory training group, meant that Odd One Out trials correct scores were on average 9.70 points lower (effect size *f*^2^ = 0.93) at post-intervention and 9.04 points lower (*f*^2^ = 0.90) at 9-month follow-up.

#### Summary

The working-memory intervention led to substantial and significant gains in performance for those in the working-memory training group on two directly trained tasks, Listening Recall and Odd One Out span, with very large effect sizes. These findings were present at both time points and were found even after controlling for pre-intervention performance and age.

### 3.4. Far-Transfer Effects

We next assessed whether there were far-transfer effects to Sentence Comprehension and Receptive Grammar skills, either immediately post-intervention or at 9-month follow-up. As four separate regression analyses were conducted (two far-transfer variables, two time points), Bonferroni corrections were made to the significance levels for the overall models (*p* < 0.01). See Table 4 for summary regression statistics and Figure 2 for graphs illustrating pre-intervention, post-intervention and 9-month follow-up scores for each group.

For post-intervention Sentence Comprehension, at step one, pre-intervention scores and age accounted for a substantial 68.5% of the variance, with only pre-intervention score a significant individual predictor. Of particular interest was the effect of adding group in step two, which produced a significant change in *R*^2^ (∆*R*^2^*),* accounting for a further 10.4% of the variance. The final model was significant, *F*(3, 43) = 53.61, *p* < 0.001, predicting 78.9% of the total variance (Adj. *R*^2^ 0.774). At 9-month follow-up, pre-intervention scores and age again accounted for a substantial 66.2% of the variance at step one, with only pre-intervention score a significant individual predictor. Again, in step two, adding group produced a significant change in *R*^2^ (∆*R*^2^), accounting for a further 9.7% of the variance. The final model was significant, *F*(3, 43) = 45.08, *p* < 0.001, predicting 75.9% of the total variance (Adj. *R*^2^ 0.742).

Thus, even with our conservative approach of controlling for the substantial variance accounted for by pre-intervention scores and age, training group was a significant predictor of Sentence Comprehension both immediately and nine months following the intervention. Inspection of unstandardised beta values in Table 4 shows that, when holding age and pre-intervention score constant, being in the active control group rather than the working-memory training group meant that Sentence Comprehension scores were on average 3.98 points lower (effect size *f*^2^ = 0.12) at post-intervention and 3.60 points lower (*f*^2^ = 0.11) at 9-month follow-up. The gains after training approached a medium effect size. If we consider the average pre-intervention Sentence Comprehension score in our working-memory training group (raw score = 21), together with their average age (8 years 0 months), a raw score of this value gives a scaled score of 6 according to the manual [55]. An increase of 4 (raw score) points over the control group at post-intervention following training would result in a raw score of 25, with an increase in the scaled score from 6 to 9 (a jump from 9th to 37th percentile).

For post-intervention Receptive Grammar, pre-intervention scores and age accounted for 54% of the variance in post-intervention scores at step one, with pre-intervention score a significant individual predictor. In step two, adding group did not produce a significant change in *R*^2^ (∆*R*^2^), accounting for only a further 2.5% of the variance. The final model was significant, *F*(3, 43) =18.59, *p* < 0.001, predicting 56.5% of the total variance (Adj. *R*^2^ 0.534). At 9-month follow-up, pre-intervention scores and age accounted for 58.1% of the variance at step one, with pre-intervention score a significant individual predictor. In step two, adding group, failed to produce a significant overall change in *R*^2^ (∆*R*^2^), accounting for a further 3.5% of the variance. The final model was significant, *F*(3, 43) = 22.93, *p* < 0.001, predicting 61.5% of the total variance (Adj. *R*^2^ 0.589).

The training group was not a significant predictor of Receptive Grammar at post-intervention or 9-month follow-up, although children in the working-memory training group did obtain numerically higher scores. Inspection of unstandardised beta values in Table 4 shows that, holding age and pre-intervention score constant, being in the active control group rather than the working-memory training group meant that blocks passed scores on Receptive Grammar were on average 1.40 points lower (effect size *f*^2^ = 0.03) at post-intervention and 1.50 points lower (*f*^2^ = 0.04) at 9-month follow-up (neither group difference was significant). In the latter case, however, there was a marginally significant effect of group (*p* < 0.055) with a small effect size.

#### Summary

The working-memory intervention led to significant gains for those in the working-memory training group on Sentence Comprehension (three to four points in the raw score), which approached a medium effect size. These findings were evident both immediately post-intervention and at 9-month follow-up and were found even after controlling for pre-intervention performance and age. No significant training group differences were found for Receptive Grammar.

### 3.5. Near-Transfer Effects (Untrained Measures of Working Memory)

Also of interest was the possibility of near-transfer effects to related working-memory tasks that were not directly trained, both immediately after the intervention and at 9-month follow-up. We assessed four short-term working-memory measures (Digit Recall, Word List Recall, Block Recall, and Pattern Span) and two executive working-memory measures (Counting Recall and Backwards Digit Recall). As 12 separate regression analyses were conducted (six working-memory variables, two time points), Bonferroni corrections were made to the significance level for the overall models (*p* < 0.004). See Table 5 for regression summaries for the short-term memory measures and Table 6 for regression summaries for the executive working-memory measures. See also Figure 2 for graphs illustrating pre-intervention, post-intervention and 9-month follow-up scores for each group.

For the four short-term working-memory measures, all regression models at post-intervention and 9-month follow-up were significant (*F*s(3, 43) ranged between 20.24 and 54.00, all *p*s < 0.001). Pre-intervention scores and age accounted for between 40.6% and 67.4% of the variance at step one in all eight models, and only pre-intervention scores were significant individual predictors in each case. At step two, adding group produced a significant change in *R*^2^ (∆*R*^2^) in all eight models, accounting for additional variance of between 10.8% and 24.0%. Being in the working-memory training group was associated with significantly higher scores on all four short-term memory assessments at both time points. The total variance accounted for in the models ranged from 58.5% to 79.0%.

Group differences associated with being in the working-memory training group were medium to medium-large across all near-transfer short-term memory measures, even after controlling for pre-intervention scores and age. Inspection of unstandardised beta values in Table 5 shows that, holding age and pre-intervention score constant, being in the active control group rather than the working-memory training group meant that trials correct scores on short-term working-memory tasks were on average between 3.73 and 5.06 points lower at post-intervention (*f*^2^ = 0.22, 0.24, 0.14, and 0.13 for Digit, Word List, Block and Pattern) and between 3.90 and 6.29 points lower at 9-month follow-up (*f*^2^ = 0.31, 0.30, 0.20, and 0.12 for Digit, Word List, Block and Pattern).

For the two untrained executive working-memory measures, all regression models at post-intervention and 9-month follow-up were significant (*F*s(3, 43) between 36.87 and 56.15, all *p*s < 0.001). Pre-intervention scores and age accounted for between 62.2% and 71.0% of the variance at step one, and only pre-intervention scores were significant individual predictors in each case. At step two, adding group produced a significant change in *R*^2^ (∆*R*^2^) in all models, accounting for additional variance of between 7.5% and 12.9%. For both EWM tasks at both time points, being in the trained group was associated with significantly higher scores. The total variance accounted for in these models ranged from 72.0% to 79.6%.

Group differences associated with being in the working-memory training group were medium to medium-small for the near-transfer executive working-memory measures, even after controlling for pre-intervention scores and age. Inspection of unstandardised beta values in Table 5 shows that, holding age and pre-intervention score constant, being in the active control group rather than the working-memory training group meant that trials correct scores on Counting Recall and Backwards Digit Recall were on average 5.44 and 3.38 points lower respectively at post-intervention (*f*^2^ = 0.15 for Counting Recall and *f*^2^ = 0.08 for Backwards Digit Recall); and 5.34 and 3.17 points lower at 9-month follow-up (*f*^2^ = 0.15 for Counting Recall and *f*^2^ = 0.08 for Backwards Digit Recall).

#### Summary

The working-memory intervention led to significant gains for those in the working-memory training group on all six untrained working-memory tasks, encompassing both short-term and executive working memory, usually with medium effect sizes. This provided strong evidence for near transfer to similar working-memory tasks. These findings were evident both immediately post-intervention and at 9-month follow-up and were found even after controlling for pre-intervention performance and age.

## 4. Discussion

This study investigated whether an adaptive intervention targeting executive working memory would benefit children with developmental language disorder, as evidenced by improvements not only on the two trained tasks (direct effects) and six untrained working-memory tasks (near-transfer effects), but also on tests of Sentence Comprehension and Receptive Grammar (far-transfer effects). Outcomes were measured immediately post-intervention and nine months later. To specifically evaluate the contribution of an executive working-memory training intervention, the study randomly assigned participants to a ‘trained’ group who received the executive verbal and visuospatial tasks (Listening Recall and Odd One Out) and an ‘active control’ group who received the same tasks but without the executive element. Hierarchical multiple regression analyses controlling for pre-intervention performance and age found group to be a significant predictor of Sentence Comprehension at both time points. These findings indicated that children in the working-memory training group obtained significantly higher scores on Sentence Comprehension than children in the active control group immediately following training, and that these gains were maintained nine months later. We estimated how the intervention affected the children’s percentile scores on Sentence Comprehension. Calculations suggested that the intervention could result in a change from the 9th to 37th percentile, an appreciable gain, as after the intervention scores would reach the low-typical range. Performance on Receptive Grammar, however, did not show these gains, with only a marginal group effect at 9-month follow-up.

In terms of direct and near-transfer effects, group was a significant predictor of performance on both the trained executive working-memory tasks (Listening Recall and Odd One Out) and performance on all other near-transfer working-memory tasks (Digit Recall, Word List Recall, Block Recall, Pattern Span, Counting Recall and Backward Digit Recall) immediately post-intervention and nine months later. For all tasks, children in the working-memory training group obtained significantly higher scores than children in the active control group at both time points, even after controlling for pre-intervention performance and age.

The findings of positive near and far-transfer effects of the working-memory training in this study run counter to earlier systematic reviews that have concluded that working-memory interventions fail to produce far-transfer effects. However, these reviews were mostly based on studies of children with TD [23,24,25,26,27]. In contrast, our findings add to more recent studies reporting far transfer to language performance, usually with children who have developmental conditions such as language difficulties [29,35,36,37,38,39]. It is notable that the present study achieved these positive outcomes following training on just two executive working-memory tasks (one verbal and one visuospatial) administered in 18 sessions of around 10 min three times a week (total 3 h). Other studies of children with DLD deployed more intervention tasks and included both short-term memory and executive working-memory components (e.g., five computer-based short-term and executive working-memory tasks in the Magic Memory programme [38,39] or multiple live memory tasks [35]; and total intervention times ranging from 12–18 h across studies). The present study is also unique in conducting a longer-term follow-up and finding that gains were retained nine months after the short, targeted intervention.

How might we account for the positive near and far-transfer effects of this intervention? Face-to-face delivery was used on the grounds that social engagement may increase children’s motivation and focused attention to input, providing favourable conditions for take-up of the training. This may be particularly important for the executive-load element of EWM tasks that the trained group received, differentiating this from the judgement subtasks that the active control group received. Indeed, children in the present study enjoyed taking part, all willingly completing the 18 sessions, and children in the trained group were keen to improve their previous score in both tasks, in effect competing with themselves. However, far transfer to language performance has also been observed in recent studies using computer-based training that, like the present training, appears to turn relatively pure working-memory tasks into engaging activities [38,39]. This suggests that the focus and/or enjoyment of the activity and motivation rather than mode of delivery may be a key factor in positive outcomes. In addition, the adaptive nature of the executive task, ensuring children are largely successful, while encouraging them to perform just above their current level of success, may be an important factor in their engagement and motivation and is noted by many authors as a key ingredient for the success of working-memory interventions [38,39].

The effect of group on performance both post-intervention and nine months later indicates that the EWM element was the active ingredient in the intervention, since the active control group experienced the same input and judgement task as the working-memory training group but without the executive load that also required recall of the final word in each sentence (verbal intervention) or location of the ‘odd one out’ (visuospatial intervention). This outcome is in line with the prediction that an EWM training would benefit language comprehension on the grounds that both cognitive activities require simultaneous processing and storage of information [46,47]. In sentence comprehension, words need to be retained in short-term memory while also processing their meaning and the meaning of the sentence. Furthermore, most items in the Sentence Comprehension task used in this study required the child to select from a set of pictures the one that matched a spoken sentence (apart from the final, most difficult items that required inferencing from a sequence of sentences presented with pictorial support). To succeed, the child must not only recognise, understand, and retain all elements of the verbal input, but must at the same time scan the visual input to find the picture that depicts all elements of the sentence, avoiding the distraction of pictures that share some but not all of these elements. The implication is that the intensive experience of simultaneous processing and storage in the training tasks improved executive management, as evidenced by the post-training gains on these tasks, to the benefit of the executive-loaded Sentence Comprehension.

It is notable that the other measure of far transfer to language comprehension (Receptive Grammar from the Test for the Reception of Grammar, TROG-2 [56]), did not show significant improvement in the working-memory training group, although there was a trend in this direction at 9-month follow-up. On our proposed interpretation of training effects, we might infer that the gains in executive management following training were not sufficient to meet the executive demands of the TROG-2 verbal/visual material, or that the TROG-2 includes grammatical structures that children have not acquired. These possibilities are plausible since the blocks of items testing grammatical constructs on the TROG-2 become increasingly complex both conceptually and syntactically. Another possibility is that the very stringent scoring on the TROG-2, with children only receiving a point for a grammatical construct if they respond correctly to all four items in the block targeting that construct, raised the bar for achieving statistically significant changes in scores.

The attribution of gains in Sentence Comprehension to improved EWM is in line with the account of training-induced changes in working memory put forward by Gathercole et al. in a systematic review of working-memory training effects [60]. According to their account, such changes arise from ‘novel cognitive routines that control the sequence of cognitive processes required to perform the task’, and far-transfer effects only arise where the directly trained task yields a new cognitive routine that can be profitably applied to a far-transfer task. However, such routines will not benefit tasks for which well-established processes are used, amongst which Gathercole et al. [60] include verbal short-term memory (STM) tasks that rely on phonological coding. This notion of ‘new cognitive routines’ receives some support from the overt strategies that some children in this study developed and used in the Odd One Out task, although the use of strategies was neither suggested nor encouraged by the researcher. One child used body parts, for example, the shoulders and head, to represent the location of left, right, and middle shapes, and performed the resulting sequence of gestures in visuospatial recall; another child tapped different fingers for different locations and used the resulting sequence of taps in recall. Strategies such as silent verbal rehearsal and chunking used in verbal recall tasks may be construed as ‘new cognitive routines’ that support recall capacity.

On Gathercole’s [60] account, near-transfer effects to non-executive (STM) tasks would also be attributed to new cognitive routines induced by the training, rather than changes in children’s basic STM capacity. The implication is that increased auditory/visual attention and/or deployment of strategies induced by the training enabled children to use their basic STM capacity more effectively (rather than directly increasing this). On this interpretation, we might expect less benefit for a nonword repetition task, which requires immediate coding and production of a novel phonological form, potentially drawing on long-term knowledge of lexical phonology, but not amenable to strategies such as rehearsal or chunking [61]. However, increases in nonword repetition have been reported following working-memory training in children with language difficulties [37], indicating that this would be an interesting avenue to explore further.

What are the wider implications of the view that EWM interventions effect change in executive control of information processing that may benefit performance on language comprehension and also non-executive working-memory tasks? Consider everyday verbal inputs that children receive, for example, a sequence of verbal instructions from a teacher, or someone recounting an event or experience. Such inputs are rapid and transitory so must be processed and interpreted ‘online’. Comprehension therefore relies on immediate verbal STM and language processing, both drawing on established language knowledge. If the training has enabled children to deploy their STM capacity more effectively, as suggested above, this may extend the amount of verbal input they can process and recognise and thereby optimise the deployment of their language knowledge in online comprehension. If the training has enhanced their capacity to store and process information simultaneously, as suggested by gains on the executive working-memory tasks, this may support better retention of processed input while processing subsequent input and thereby improve their comprehension of stretches of verbal discourse. It should particularly benefit activities that do not involve rapid and transitory input, for example, reading comprehension and school-based learning that requires close attention to and the integration of new pieces of information.

Having reported the positive outcomes of this study, it is important to acknowledge its limitations. Most notably, due to resource limitations, the sample size was moderate, and the researcher who delivered the training also administered all the assessments so was not blind to the child’s group. This may have led to researcher bias, resulting in more favourable outcomes for the trained group. It is worth pointing out that outcomes in this group varied, and that some children showed little improvement. Nevertheless, replication of the study with larger samples and blinded assessment is needed to preclude possible bias. Further research is also needed to investigate whether there are wider benefits for language performance, of the sort proposed above, in the short and longer term.

## 5. Conclusions

Working-memory training that targeted executive working-memory skills was found to have far, near, and direct transfer effects in children with developmental language disorder, not only immediately post-intervention but also nine months later. This indicates its potential as an intervention to improve working memory and language comprehension in these children. The intervention programme involved short sessions over a short period, causing little disruption in the school day, and children enjoyed the activities. The programme is relatively straightforward to administer and could be delivered by trained teaching support staff. The findings from this study invite independent evaluation with blind assessment and further research to throw more light on which children benefit, which language and other skills show immediate benefit, and whether benefits are sustained in the even longer-term.

## Figures and Tables

**Table 1:** Mean screening scores (SD) for children in the working-memory training and active control groups. Independent samples *t*-tests for group differences are also presented.

Screening Scores	Working-Memory Training Group (*n* = 24)	Active Control Group (*n* = 23)	Group Differences
Age in months	96.17 (13.96)	96.17 (16.00)	*t*(45) = −0.002, *p* = 0.999, *d* = 0.00 (−0.57, 0.57)
Non-verbal IQ (perceptual reasoning index, WASI-II) ^1^	92.04 (14.69)	91.00 (12.44)	*t*(45) = 0.26, *p* = 0.795, *d* = 0.08 (−0.50, 0.65)
Concepts and following directions, CELF-4-UK ^2^	5.12 (3.01)	3.78 (2.88)	*t*(45) = 1.56, *p* = 0.125, *d* = 0.46 (−0.13, 1.03)
Word structure, CELF-4-UK ^2^	4.75 (1.89)	3.78 (2.19)	*t*(45) = 1.62, *p* = 0.112, *d* = 0.47 (−0.11, 1.05)
Recalling sentences, CELF-4-UK ^2^	4.96 (2.56)	4.09 (2.35)	*t*(45) = 1.21, *p* = 0.231, *d* = 0.35 (−0.23, 0.93)
Formulated sentences, CELF-4-UK ^2^	3.67 (2.70)	2.52 (1.93)	*t*(45) = 1.67, *p* = 0.102, *d* = 0.49 (−0.10, 1.07)

^1^ Standardised scores (Mean = 100; SD = 15); ^2^ Scaled scores (Mean = 10; SD = 3).

**Table 2:** Mean raw scores (SDs) for pre-intervention, post-intervention and 9-month follow-up on all outcome measures for children in the working-memory training and active control groups.

Outcome Variables	Time (Pre-Intervention, Post-Intervention or 9-Month Follow-Up)	Working-Memory Training Group (*n* = 24)	Active Control Group (*n* = 23)
Listening recall, WMTB-C	Pre-intervention	9.21 (3.62)	9.52 (5.53)
	Post-intervention	16.33 (3.77)	10.00 (5.71)
	Follow-up	16.38 (4.41)	9.78 (5.13)
Odd One Out Span	Pre-intervention	9.08 (4.10)	8.78 (3.25)
	Post-intervention	18.46 (6.16)	8.52 (3.37)
	Follow-up	18.75 (5.98)	9.48 (2.98)
Sentence Comprehension, ACE	Pre-intervention	21.00 (5.85)	18.91 (6.42)
	Post-intervention	25.79 (4.37)	20.22 (6.42)
	Follow-up	27.46 (4.16)	22.39 (6.05)
Receptive Grammar, TROG-2	Pre-intervention	8.50 (3.58)	6.83 (3.51)
	Post-intervention	11.25 (4.37)	8.35 (3.73)
	Follow-up	12.13 (3.81)	9.35 (3.46)
Digit Recall, WMTB-C	Pre-intervention	25.83 (6.06)	24.57 (4.33)
	Post-intervention	31.13 (6.02)	25.26 (4.32)
	Follow-up	32.04 (6.19)	24.83 (4.51)
Word List Recall, WMTB-C	Pre-intervention	18.83 (3.64)	17.00 (2.91)
	Post-intervention	22.67 (4.13)	17.13 (3.09)
	Follow-up	23.29 (4.18)	17.22 (2.65)
Block Recall, WMTB-C	Pre-intervention	20.67 (6.40)	20.00 (5.83)
	Post- intervention	25.87 (5.90)	21.09 (5.59)
	Follow-up	26.42 (5.60)	21.26 (4.89)
Pattern Span	Pre-intervention	17.29 (5.15)	16.04 (5.51)
	Post- intervention	21.63 (5.09)	17.13 (5.06)
	Follow-up	22.46 (5.76)	17.74 (5.26)
Counting Recall, WMTB-C	Pre-intervention	16.58 (5.49)	15.22 (6.18)
	Post-intervention	22.96 (7.03)	16.30 (6.67)
	Follow-up	23.33 (6.83)	16.78 (6.62)
Backwards Digit Recall, WMTB-C	Pre-intervention	11.21 (5.66)	9.74 (5.92)
	Post- intervention	14.08 (5.74)	9.52 (5.77)
	Follow-up	13.83 (5.65)	9.57 (4.93)

**Table 3:** Summary of step 2 of the regressions for the outcome variables Listening Recall and Odd One Out (assessing the direct effects of the training intervention).

Predictors at Step 2	B (95% CIs) ^1^	SE B	β
Listening Recall post-intervention			
Pre-intervention score	0.89 (0.67, 1.11)	0.11	0.71 ***
Age	−0.02 (−0.09, 0.05)	0.03	−0.05
Group ^1^	−6.61 (−8.25, −4.98)	0.81	−0.58 ***
Listening Recall 9-month follow-up			
Pre-intervention score	0.83 (0.62, 1.03)	0.10	0.66 ***
Age	0.02 (−0.04, 0.08)	0.03	0.05
Group ^1^	−6.85 (−8.40, −5.31)	0.77	−0.60 ***
Odd One Out post-intervention			
Pre-intervention score	0.79 (0.50, 1.08)	0.15	0.41 ***
Age	0.10 (0.03, 0.18)	0.04	0.22 **
Group ^1^	−9.70 (−11.7, −7.7)	0.99	−0.70 ***
Odd One Out 9-month follow-up			
Pre-intervention score	0.79 (0.51, 1.07)	0.14	0.44 ***
Age	0.09 (0.02, 0.16)	0.03	0.20 *
Group ^1^	−9.04 (−10.92, −7.15)	0.94	−0.69 ***

*** *p* < 0.001; ** *p* < 0.01; * *p* < 0.05. ^1^ Unstandardised betas (B) for group indicate how much lower the outcome variable score was on average in the active control group compared to the trained group, holding the other variables constant (95% confidence intervals for these B-estimates are given in brackets).

**Table 4:** Summary of step 2 of the regressions for the far-transfer outcome variables (ACE Sentence Comprehension and TROG-2 Receptive Grammar).

Predictors	B (95% CIs) ^1^	SE B	β
Sentence Comprehension post-intervention			
Pre-intervention score	0.76 (0.60, 0.93)	0.08	0.77 ***
Age	−0.001 (−0.07, 0.07)	0.03	−0.002
Group ^1^	−3.98 (−5.72, −2.24)	0.86	−0.33 ***
Sentence Comprehension 9-month follow-up			
Pre-intervention score	0.70 (0.54, 0.87)	0.08	0.76 ***
Age	0.00 (−0.07, 0.07)	0.03	0.001
Group ^1^	−3.60 (−5.34, −1.85)	0.87	−0.32 ***
Receptive Grammar pre-intervention to post-intervention			
Pre-intervention score	0.89 (0.57, 1.23)	0.16	0.76 ***
Age	−0.03 (−0.11, 0.05)	0.04	−0.11
Group ^1^	−1.40 (−3.21, 0.41)	0.90	−0.17
Receptive Grammar pre-intervention to 9-month follow-up			
Pre-intervention score	0.76 (0.48, 1.05)	0.14	0.71 ***
Age	0.002 (−0.07, 0.07)	0.03	0.006
Group ^1^	−1.50 (−3.04, 0.04)	0.76	−0.20 (*p* = 0.055)

*** *p* < 0.001. ^1^ Unstandardised betas (B) for group indicate how much lower the outcome variable score was on average in the active control group compared to the trained group, holding the other variables constant (95% confidence intervals for these B-estimates are given in brackets).

**Table 5:** Summary of step 2 of the regressions for the short-term memory near-transfer outcome variables (Digit Recall, Word List Recall, Block Recall and Pattern Span).

Predictors	B (95% CIs) ^1^	SE B	β
Digit Recall post-intervention			
Pre-intervention score	0.63 (0.39, 0.88)	0.12	0.56 ***
Age	0.03 (−0.06, 0.11)	0.04	0.07
Group	−5.06 (−7.42, −2.69)	1.17	−0.43 ***
Digit Recall 9-month follow-up			
Pre-intervention score	0.73 (0.50, 0.96)	0.11	0.59 ***
Age	0.02 (−0.06, 0.11)	0.04	0.06
Group	−6.29 (−8.52, −4.06)	1.11	−0.49 ***
Word List Recall post-intervention			
Pre-intervention score	0.77 (0.53, 1.01)	0.12	0.57 ***
Age	0.03 (−0.03, 0.08)	0.03	0.08
Group	−4.12 (−5.70, −2.54)	0.78	−0.46 ***
Word List Recall 9-month follow-up			
Pre-intervention score	0.81 (0.59, 1.02)	0.11	0.59 ***
Age	0.01 (−0.04, 0.06)	0.02	0.03
Group	−4.60 (−6.01, −3.19)	0.70	−0.50 ***
Block Recall post-intervention			
Pre-intervention score	0.74 (0.55, 0.93)	0.10	0.73 ***
Age	0.00 (−0.08, 0.08)	0.04	0.00
Group	−4.30 (−6.43, −2.16)	1.06	−0.35 ***
Block Recall 9-month follow-up			
Pre-intervention score	0.68 (0.49, 0.86)	0.09	0.71 ***
Age	−0.02 (−0.09, 0.06)	0.04	−0.05
Group	−4.70 (−6.73, −2.68)	1.01	−0.41 ***
Pattern Span post-intervention			
Pre-intervention score	0.78 (0.62, 0.93)	0.08	0.76 ***
Age	0.04 (−0.02, 0.09)	0.03	0.10
Group	−3.73 (−0.5.27, −2.19)	0.77	−0.34 ***
Pattern Span 9-month follow-up			
Pre-intervention score	0.83 (0.65, 1.01)	0.09	0.75 ***
Age	0.03 (−0.03, 0.10)	0.03	0.08
Group	−3.90 (−5.70, −2.10)	0.89	−0.33 ***

*** *p* < 0.001. ^1^ Unstandardised betas (B) for group indicate how much lower the outcome variable score was on average in the active control group compared to the trained group, holding the other variables constant (95% confidence intervals for these B-estimates are given in brackets).

**Table 6:** Summary of step 2 of the regressions for the executive working-memory near-transfer outcome variables (Counting Recall, Backwards Digit Recall).

Predictors at Step 2	B (95% CIs) ^1^	SE B	β
Counting Recall post-intervention			
Pre-intervention score	0.89 (0.68, 1.10)	0.10	0.68 ***
Age	0.09 (0.004, 0.17)	0.04	0.17 *
Group	−5.44 (−7.54, −3.34)	1.04	−0.36 ***
Counting Recall 9-month follow-up			
Pre-intervention score	0.89 (0.66, 1.11)	0.11	0.69 ***
Age	0.05 (−0.04, 0.14)	0.04	0.10
Group	−5.34 (−7.62, −3.06)	1.13	−0.36 ***
Backwards Digit Recall post-intervention			
Pre-intervention score	0.81 (0.60, 1.01)	0.10	0.76 ***
Age	0.01 (−0.07, 0.09)	0.04	0.02
Group	−3.38 (−5.38, −1.38)	0.99	−0.28 **
Backwards Digit Recall 9-month follow-up			
Pre-intervention score	0.75 (0.58, 0.91)	0.08	0.76 ***
Age	0.04 (−0.03, 0.10)	0.03	0.09
Group	−3.17 (−4.78, −1.57)	0.80	−0.28 ***

* *p* < 0.05; ** *p* < 0.01; *** *p* < 0.001. ^1^ Unstandardised betas (B) for group indicate how much lower the outcome variable score was on average in the active control group compared to the trained group, holding the other variables constant (95% confidence intervals for these B-estimates are given in brackets).

## Data Availability

The data presented in this study are available on request from the corresponding author. The data are not publicly available due to the nature of consent provided by participants.
